# Antiseptic Surgery

**Published:** 1891-02-28

**Authors:** 


### SURGERY.

### ANTISEPTIC SURGERY.

It is a humiliating fact that at this stage of its history the practice of antiseptic surgery should have to be defended. It had seemed as if no controversy could shake the system which the genius of Lister has given to the world; but now this faith is rudely shaken, and the signs of wavering are widely apparent. To what are we to attribute this "falling off"? Mainly, we think, to two sources—the attacks of its opponents, and still more, perhaps, to a misunderstanding of the characteristic address given by Sir Joseph Lister himself at the Berlin Congress. That this last could happen shows how loose a hold the essential principles of antiseptic surgery had in many cases; the details have been confounded with the principle—the particular form of dressings recommended at the time, with the fundamental idea expressed by them; and to make "confusion worse confounded," the array of these details has been, in mistaken honour, christened "Listerism," so that some, seeing the arrangement and composition of these details subject to much change, have imagined that the thing itself was changing, and therefore had no foundation on which to rest. Surely it would have been a far more splendid and truthful honour (and it would have saved much confusion) to have given the name of "Listerism" to any form of dressing or treatment that efficiently carried out the great principles that Lister preached. We should then have heard no pitiful (or triumphant) wailings over the removal of this or that item of the antiseptic treatment, and the grand principles underlying it would have shone the clearer from the very vicissitudes of its outward form.

Lister says, "As regards the spray, I feel ashamed that I should have ever recommended it *for the purpose of destroying the microbes of the air*" (the italics are ours). The first part is quoted triumphantly without its context, and no notice is taken of the reasons given for abandoning its use. Because Lister finds that the spray does not purify the air as he thought it did, and thinks that its virtue as a constant irrigator does not counterbalance the irritation it produces, and so gives up using it, this surely is no death-blow to his system. That it can be so regarded is only another proof of a weak grasp of principles. The great influence exerted by the living tissues in checking bacteric development, although long ago pointed out and proved by Lister, has not always been rated at its due value in analyzing the results of conflicting systems of wound treatment. It serves to explain many happy escapes from imperfect applications of the antiseptic method, as well as the behaviour of wounds where this method cannot be employed.

Lately this vital influence has been traced by Metchnikoff to wandering amœboid cells, which devour the microbes and destroy them by intracellular digestion. To these cells—which are in fact leucocytes (whether all or only special kinds cannot be said)—he has given the name of "phagocytes," and their presence has been demonstrated experimentally in ordinary inflammatory effusion. Tchistovitch and Armand Ruffer have verified and extended his observations so that they may be considered as fairly established. But it is surely a false deduction (false at least in *practice*) to say that as the body possesses this army of guard-cells, we need take no thought for the germs we admit to our wounds. Again, the all-importance of the pabulum as a feeding-ground for germs has been magnified to the exclusion of all other considerations. Leave no pabulum, say some, and then what about your germs? There is nothing more dangerous than a theory, however sound, that will not fit the exigencies of every-day practice. The argument would be absolutely final (and would in no respect clash with all that Lister has taught) could it be carried out in practice. It means, as Professor Lister said in his Berlin address, a condition of wound, where there will be no more exudation than the surrounding tissues can themselves dispose of; where no irritation will arise from drainage-tubes or dressings; where the wound, in short, will be like a subcutaneous injury, and the disturbing influence of germs is out of court. This, indeed, is a "consummation devoutly to be wished," but we make bold to say that, even in its limited application to surgical wounds, it is at present very far from possible to the majority of us. We cannot get rid of the pabulum of blood and serum sufficiently to justify our ignoring the germs in the majority of cases. It has, indeed, been argued with curious disregard of logic, that germs are ever present in the blood and so cannot be got rid of, and yet, at the same time, a broad-ligament hæmatocele has been given as an instance of a subcutaneous effusion which does not decompose *because* germs have no access! How is this, if germs cannot be got rid of because they are ever present? It is noteworthy that the greatest amount of opposition to the antiseptic method comes from some abdominal surgeons. Their experience, however, is not by any means a safe example in ordinary surgical cases. The peritoneum is a huge lymph-space, consequently the absorption of the serum effused from an operation (pabulum) is much more speedily absorbed than in most other parts, and, when present, probably contains a very large number of leucocytes or "phagocytes"; secondly, the peritoneal incision is soundly closed against the air in about 24 hours, so that we have only to reckon with possibilities of infection for this space of time.

We are told that the peritoneal cavity is flushed with ordinary water; the peculiar protective power of the peritoneal sac must stand it in good stead, for no one, we think, would venture on the same treatment in the case, say, of a joint. But even these surgeons are most particular that everything used during the operation should be scrupulously cleansed, and sponges in particular. How this is done is not of much consequence, although we have no doubt of the superiority of germicidal solutions for routine practice; but to assert that a dip in a 5 per cent. carbolic solution is supposed to cleanse anything is a statement that only the laxity of some so-called antisepticians could make for a moment credible. But, after all, antiseptic surgery has even more solid ground to stand on—the comparative statistics with and without its precautions. Take Lister's own statistics at Glasgow from 1864 to 1869. His mortality for amputations for 1864 and 1866 was 45·7 per cent.; from 1867 to 1869, with aseptic treatment, it was 15 per cent. These are results by the same surgeon in the same hospital and under identical conditions. Again, in Edinburgh, Professor Lister had two series of cases. Some were treated aseptically, with a mortality from septic disease of ·36 per cent.; others were treated variously, with antiseptics and without, and of these 1·36 per cent. died from the same cause, or a mortality four times as great as in the first. Compare with these Spence's statistics of operations in the same hospital not treated aseptically. These give a general mortality of nearly 18 per cent. against 5 per cent. in Professor Lister's cases, and a mortality from septic diseases, as far as is known (though probably it should be much larger), of 2·4 per cent., or eight times greater than Lister's. And if we turn to foreign testimony it is even more striking. Volkmann was just going to shut up his hospital on account of the number of deaths from pyæmia and septicæmia, when he tried Lister's method. Amongst cases treated aseptically there was one death from pyæmia and one from erysipelas! All other conditions remained the same; the method of treatment only was altered. And testimony of the same value is afforded by the statistics of numerous observers too many to particularise, but the names of Nussbaum, Saxtorph, Esmarch, Czerny, Hueter, and Létiévant may be given as some of those who have found their results revolutionised by the antiseptic treatment. Full particulars of these can be found in Mr. Cheyne's book.

